# Diagnosis and treatment of obturator hernia: retrospective analysis of 86 clinical cases at a single institution

**DOI:** 10.1186/s12893-021-01125-2

**Published:** 2021-03-09

**Authors:** Zhengzheng Li, Chaoyang Gu, Mingtian Wei, Xing Yuan, Ziqiang Wang

**Affiliations:** Department of Gastrointestinal Surgery, West China Hospital, Sichuan University, No. 37 Guo Xue Alley, Chengdu, 610041 Sichuan China

**Keywords:** Obturator hernia, Diagnosis, Intestinal necrosis, Surgical treatment, Mortality, Recurrence

## Abstract

**Background:**

To explore the clinical characteristics, diagnosis and treatment of obturator hernia.

**Methods:**

Eighty-six patients who were diagnosed as obturator hernia by abdominal CT in the Department of Gastrointestinal Surgery of our hospital between 2009 and 2019 were enrolled in this study. Patient characteristics, surgical method, postoperative complications and mortalities were retrospectively reviewed.

**Results:**

Thirty days mortality rate of 5.5% and 46.1% were observed in surgery group and non-surgery group, respectively. Surgery was performed as an emergency procedure in 59 cases and elective procedure in 14 cases depending on different hernia contents, intestinal necrosis and signs of peritonitis. In the emergency surgery group, segmental intestinal resection with anastomosis was performed in 24 patients (24/59, 40.7%). There were 4 deaths (4/59, 6.8%) in this group, all of which occurred in patients undergoing SI resections. In contrast, no bowel resection, postoperative complications, or death occurred in the elective surgery group. 3-year recurrence rates of 5.1% (3/59) and 7.1% (1/14) were observed in the emergency surgery and the elective surgery group, respectively.

**Conclusions:**

CT examination plays an important role in improving the diagnostic rate of obturator hernia. Timely surgical treatment is the key to improve the efficacy of obturator hernia and prevent the deterioration of the condition. In addition, intestinal resection and postoperative complications may be the important factors leading to postoperative death.

## Background

As a rare type of abdominal hernia and first described by Ronsil in 1724, obturator hernia (OH) is caused by herniation of intra-abdominal contents through obturator foramen [1]. Although OH accounts for only 0.05 to 1.4% among all the hernias [2], it is a challenge for diagnosis due to lack of clinical features and unclear expression of their conditions of the elders. OH normally presents as a significant cause of intestinal obstruction especially in emaciated elderly women. Richter’s type is the most prevalent type in OH, which can develop into necrosis with or without obvious intestinal obstruction [3, 4].The mortality of OH is relatively high especially for strangulated cases, ranging from 12 to 70%[5]. Accordingly, early diagnosis and timely surgical intervention are critical. The obturator foramen, which is the largest foramen in the body, is sealed by a thick membrane and drilled by the obturator canal. The canal is 2 to 3 cm long and has obturator nerves and vessels passing through it which are covered peripherally by adipose tissue. The loss of this fatty tissue, which is caused by cachexia or malnutrition, will increase the risk for the occurence of obturator hernia [6–8]. Additional predisposing factors include conditions such as constipation, multiparity, ascites, chronic obstructive pulmonary diseases and other conditions which can increase intra-abdominal pressures [9–11].

As usual, OH patients are elderly emaciated females with many comorbidities, which bring high risk for surgical intervention and postoperative complications. As a result, clinicians may prefer conservative treatment. However, for most cases emergency operations are necessary because the patients already had severe intestinal obstruction and even intestinal necrosis due to prolonged incarceration, leading to peritonitis. The percentage of strangulation can reach 50–75% [12]. Here we reviewed 86 cases of obturator hernia patients who were diagnosed and treated in our hospital in order to shed more lights for this rare disease.

## Methods

**Fig. 1:**
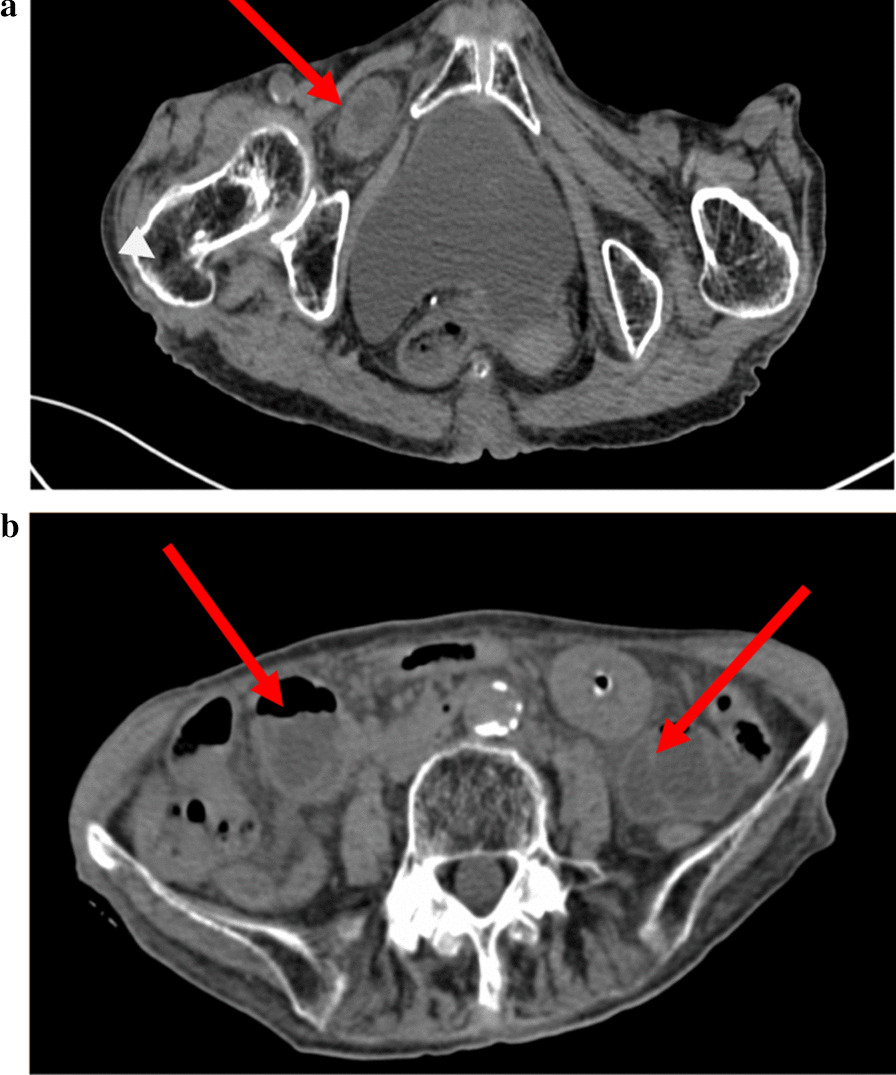
**a** Abdominal CT showing small bowel herniated into the right obturator canal. **b** Abdominal CT showing the dilated small bowel loops above the site of obstruction

**Fig. 2:**
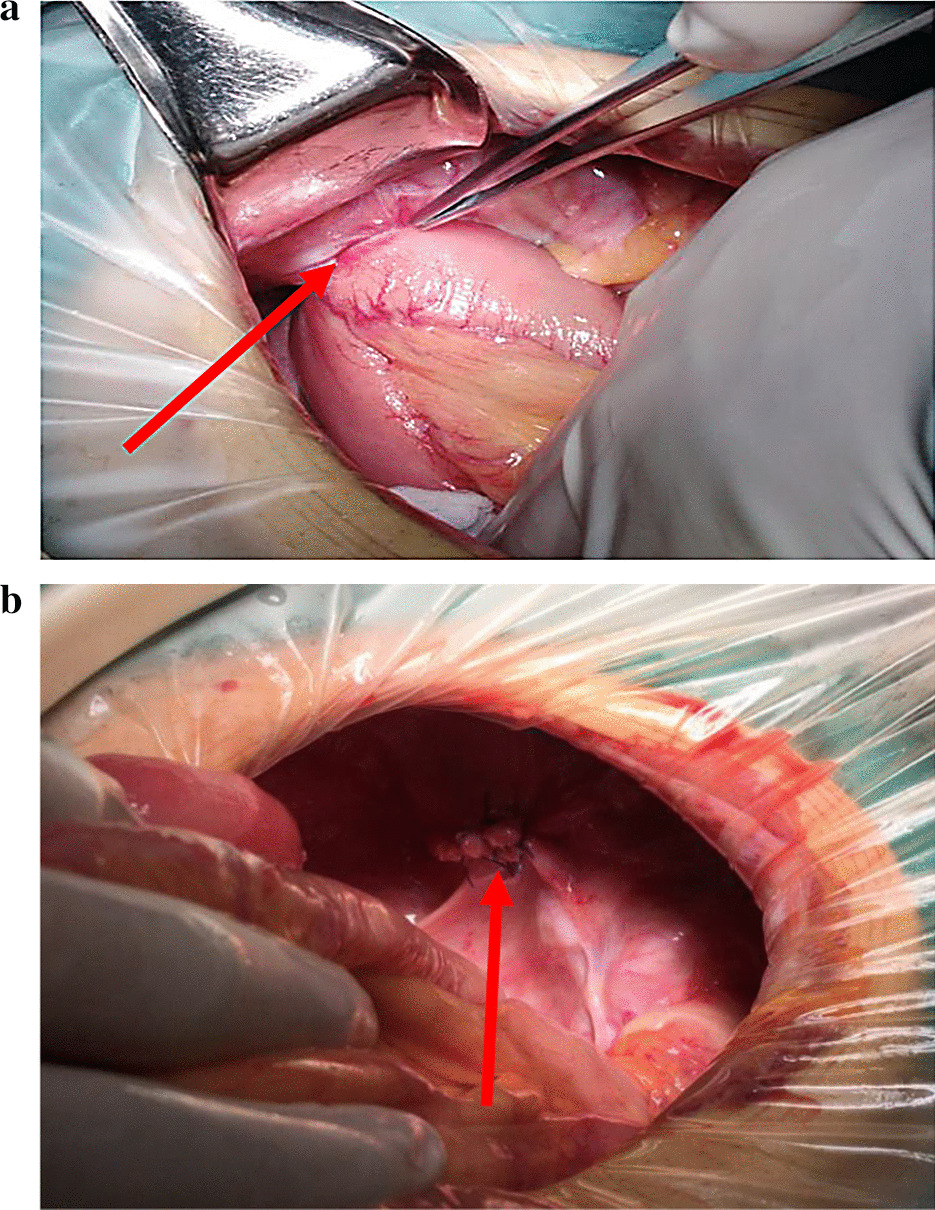
**a** Intraoperative image showing a part of the bowel protruding through the right obturator foramen. **b** Intraoperative image showing the obturator foramen after intermittent suture

We selected "obturator hernia" as the key word to search from the patient database of West China Hospital of Sichuan University. The medical records of 86 patients who were diagnosed as obturator hernia from January 2009 to September 2019 were retrieved and retrospectively reviewed. All the patients was diagnosed as obturator hernia based on preoperative computed tomography (CT) findings (Fig. 1a). Of the 86 patients, 73 patients underwent surgical treatment (surgery group) and 13 patients abandoned surgical treatment (non-surgery group) because of underlying diseases, poor general condition, high surgical risk and financial difficulties. Surgery was performed as a elective procedure in 14 (19%) and as an emergency procedure (within 24 h of consultation) in 59 (81%) cases depending on different hernia contents, presence of intestinal obstruction, intestinal necrosis and signs of peritonitis or not. Emergency surgery was performed through a lower midline incision and primary repair with simple closure and apposition of the peritoneum (Fig. 2b). We didn’t use mesh to seal the hernia orifice in case of infection. Other patients opted for elective surgery, usually with a inguinal incision and use of the mesh for repair. Patients’ characteristics, operating time, surgical method, length of hospital stay, postoperative complications and mortality rate were retrospectively reviewed. We informed patients by telephone to come to our hospital for follow-up, and conducted abdominal physical examination and abdominal CT examination of these patients in the outpatient clinic at 3 years after discharge.

Categorical variables are expressed as counts (%) and median (range) while continuous variables are expressed as mean ± standard deviation (SD). Chi square test was used to compare categorical variables, independent t-test was used to compare continuous variables. Data analysis was performed by SPSS (version 20.0, IBM Inc., Armonk, NY). Statistical significance was set at a p value < 0.05. This study was approved by Biomedical Ethics Committee of West China Hospital, and written informed consent from patients was waived because of its retrospective design.

## Results

### Patients demorgraphics

This obturator hernia series comprises of 81 females and 5 males. Patients were divided into surgery group and non-surgery group. According to the Table 1, we can see that obturator hernia is prevalent in the elderly, thin, multiparous and chronically illed women with many comorbidities. Peritonitis was discovered in 10 (13.7%) and 2 (15.4%) patients in surgery and non-surgery group, respectively. 24 (32.9%) patients and 2 patients (15.4%) had undergone prior abdominal surgery from these two groups of patients. 55 patients (75.3%) from surgery group and 6 patients (46.2%) from non-surgery group had a prior history of symptoms and signs of intestinal obstruction. The Howship–Romberg sign was positive in 10 cases (13.7%) and 1 case (7.7%) in surgery and non-surgery group, respectively. 23 patients out of 73 patients (31.5%) who accepted surgery after diagnosis were transferred to ICU. The mean duration in ICU was 5.57 ± 4.71 days with variation from 1 to 22 days. We also called the 13 patients in the non-surgery group to come to our clinic for the follow-up. 6 patients who had been diagnosed with intestinal necrosis died within 30 days, and 3 patients had surgery in other hospitals with good postoperative results. As for the mortality in 30 days, 5.5% in surgery group and 46.1% in non-surgery group was observed, which shows statistically significant difference between two groups.

**Table 1:** Demographics of patients enrolled in this study

Patient characteristics	Surgery Group	Non-surgery Group	*p* value
Gender			
Male/female	2/71	3/10	0.004
Age (mean ± SD, range)	79.47 ± 8.84 (59–103)	78.69 ± 7.63 (64–93)	0.43
BMI (mean, range)	16.78 ± 3.0 (11.38–29.03)	20.17 ± 2.28 (17.71–22.20)	0.062^a^
Side at initial diagnosis n (%)			0.719
Left	31 (42.5)	6 (46.2)	
Right	30 (41.1)	6 (46.2)	
Bilateral	12 (16.4)	1 (7.7)	
Number of deliveries (mean ± SD, range)	4.86 ± 2.64 (1–12)	5.75 ± 2.22 (3–8)	0.502
Intestinal obstruction n (%)	55 (75.3)	6 (46.2)	0.033
Peritonitis n (%)	10 (13.7)	2 (15.4)	0.872
Howship–Romberg sign n (%)	10 (13.7)	1 (7.7)	0.550
History of abdominal surgery n (%)	24 (32.9)	2 (15.4)	0.206
Transfer to ICU n (%)	23 (31.5)	0 (0)	0.018
Days of stay in ICU (mean, range)	5.57 ± 4.71 (1–22)	–	–
Readmission n (%)	5 (6.8)	0	0.431^b^
Mortality in 30 days n (%)	4 (5.5)	6 (46.1)	0.004

### Outcomes of the emergency and elective surgical treatment

**Fig. 3:**
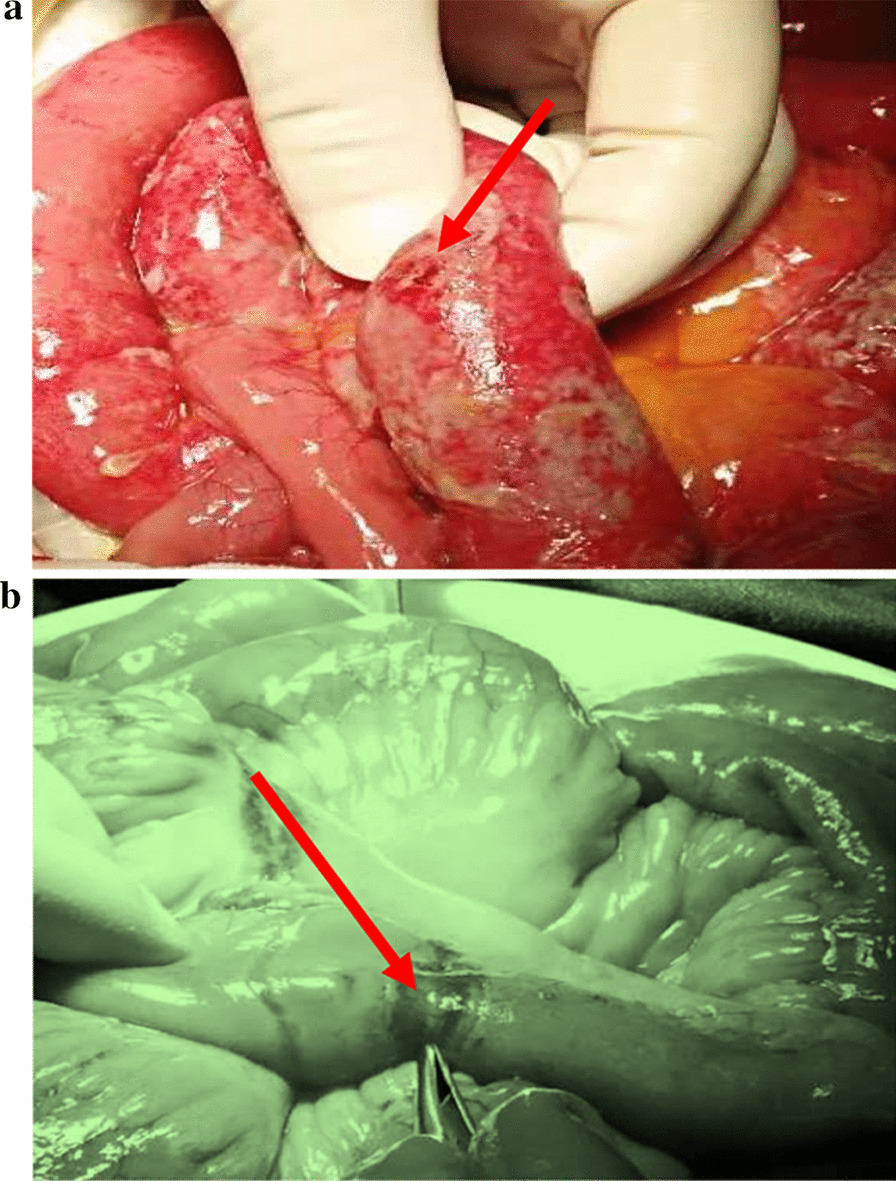
**a** Intraoperative image showing the intestinal perforations caused by obturator hernia. **b** Intraoperative image showing the intestinal necrosis caused by obturator hernia

As summarized in Table 2, the outcomes of the 73 patients who received surgical treatment were listed. For emergency group: the mean duration from appearance of symptoms to surgery was 3 days (range 1–9). The hernia contained small bowel in 51 cases (86.4%) and the rate of strangulated hernias was 74.6% (44 cases). Segmental intestinal resection with anastomosis was performed in 24 patients (40.7%), who had intestinal perforations (Fig. 3a) or necrosis (Fig. 3b). Postoperative complications occurred in 4 patients (4/59, 6.8%),all of which were in patients undergoing SI resections and eventually resulted in death in this data set. One patient died of MODS, and one patient died of AECOPD. The other two patients died of pneumonia. No anastomotic leakage and surgical site infection (SSI) were detected in this cohort of patients. The recurrence rate at 3 years after the initial obturator hernia surgery was 5.1% (3/59 cases). For elective group: the mean duration from the appearance of symptoms to surgery was 7.5 days (range 3.0–365). The inguinal approach was selected and primary repaired with mesh in 14 operations (100%). The hernia contained fat in 8 cases (57.1%) and small bowel in 2 cases (14.3%). No patient had postoperative complications nor deaths or surgical site infection (SSI) in this group. The recurrence rates at 3 years after the surgery was 7.1% (1/14 cases).

**Table 2:** Outcomes of the emergency and elective surgical treatment (n = 73)

Parameter	Emergency (n, %)	Elective (n, %)	*P*
ASA grade			
II	22 (37.3)	5 (35.7)	> 0.999
III	36 (61.0)	9 (64.3)	
IV	1 (1.7)	0 (0.0)	
Duration from appearing symptoms to surgery (days,media, range)	3 (1.0–9.0)	7.5 (3.0–365.0)	0.038^c^
Surgery approach			
lower midline incision	59 (100)	0 (0.0)	
Inguinal incision	0 (0.0)	14 (100)	
Operative time (minutes, mean ± SD, range)	101.4 ± 28.9 (60–180)	68.6 ± 17.0 (60–120)	< 0.001^b^
Incarcerated n (%)	44 (74.6)	0 (0.0)	-
Contents in hernia sac			< 0.001
Nothing	0 (0.0)	2 (14.3)	
Small intestine	51 (86.4)	2 (14.3)	
Omentum	2 (3.4)	2 (14.3)	
Mesentery of intestine	3 (5.1)	0 (0.0)	
Fat	3 (5.1)	8 (57.1)	
Contralateral concealed obturator hernia			< 0.001
Yes	10 (16.9)	1 (7.1)	
No	46 (78.0)	6 (42.9)	
Bilateral at initial diagnosis	3 (5.1)	7 (50.0)	
Hernia in other sites (Groin, Femoral, Lumbar)	5 (8.5)	1 (7.1)	0.676
n (%)			
Intestinal resection n (%)	24 (40.7)	0 (0.0)	–
Primary repair n (%)	59 (100.0)	14 (100.0)	–
Blood loss (ml, mean ± SD, range)	21.3 ± 22.7 (5–100)	9.6 ± 5.4 (5–20)	0.013
Days of antibiotics administration (mean ± SD, range)	5.3 ± 4.0 (0–24)	4.4 ± 7.8 (0–30)	0.565
Intraoperative complications	0 (0.0)	0 (0.0)	–
Postoperative complications	4 (6.8)	0 (0.0)	–
Pneumonia	2	–	
AECOPD	1	–	
MODS	1	–	
Clavien-Dindo Grade			–
I–II	1 (1.7)	–	
III–IV	1 (1.7)	–	
V	2 (3.4)	–	
Postoperative hospital stay (days, mean, range)	8.3 ± 5.3 (1–31)	7.3 ± 6.8 (1–30)	0.534
Total Length of hopstital Stay	9.3 ± 5.3 (2–32)	8.4 ± 6.8 (2–31)	0.576^b^
Mortality in 30 days	4 (6.8)	0 (0.0)	–
Recurrence	3 (5.5)	1 (7.1)	0.093^a^
Length from discharge to recurrence (months, media, range)	12 (6–20)	5 (6–24)	–

*ICU* intense care unit

^a^Fisher exact test, ^b^ student t test, ^c^ Mann–Whitney U test

## Discussion

This is a retrospective analysis of the clinical treatment of patients with obturator hernia over a 10-year period in a single center. Patient characteristics, operation time, surgical method, length of hospital stay, postoperative complications and mortalities were retrospectively reviewed. The patients’ characteristics in this study were consistent with previous reports that obturator hernia is a scare type of abdominal hernia with the nickname "little old lady's hernia". It is prevalent in the elderly, chronically illed, multiparous and thin women with many comorbidities [10, 13]. Small bowel is the most common content in the hernia sac, and can also be appendix, omentum, ovary, Meckels diverticulum, fallopian tube and even uterus [14, 15]. The diagnosis and treatment are usually delayed until laparotomy due to bowel obstruction, necrosis or inperitonitis which always lead to a high mortality [16–18]. Rectal examination and bloods are often unremarkable. Plain abdominal X-rays usually only reveal bowel obstruction or perforation. However, from the typical CT scan we can see protrusion of bowel or fat through the foramen between the pubic muscle and obturator externus muscles. And in strangulated cases, CT scan also could show the distent intestine loops and air-fluid plane. Moreover, in serious cases, the edema, ischemia, necrosis and perforation of intestine could be detected by CT. Indeed, the diagnostic capabilities of CT and MRI have become increasingly accurate [19, 20]. In fact, a multidetector CT scan including pelvis has improved the preoperative diagnosis rate to 90% [21] since it was used to detect obturator hernia by Meziane et al. [22, 23]. In our study, all the 86 patients (100%) had been diagnosed as obturator hernia based on preoperative computed tomography (CT) findings which provided us with great help and modified our preoperative misdiagnosis of inguinal hernia. A typical CT scan was shown in Fig. 1. Typical small bowel herniated into the right obturator canal (Fig. 1a) and dilated small bowel loops above the site of obstruction (Fig. 1b) were shown. Although Howship-Romberg sign is a definite indicator for obturator hernia [24, 25], it was positive only in 11 cases (12.5%) in this study. According to some previous reports, OH is more likely to occur on the right side due to the fact that sigmoid colon locates at the left-side of the pelvis [26, 27]. However, in our retrospective analysis of 86 patients with OH, there was no significant difference in incidence between the left and right sides.

The difference in 30-day mortality between the surgical and non-surgical groups had provided strong evidence that surgery was the best way to treat the disease. Moreover, obturator hernia repair had traditionally been performed with a lower midline incision approach. Recently, the laparoscopic approach has been reported as a minimally invasive technique and inguinal approach with mesh repair has also been introduced as less invasive treatment. However, if the patient’s general condition is poor (presence of intestinal obstruction, intestinal necrosis or perforation, signs of peritonitis and severe comorbidities), immediate release of intestinal obstruction or segmental bowel resection must be performed with a midline incision under general anesthesia. In such cases, primary closure without prosthetic materials can be chosen in case of a high risk of infection. In the emergency surgery group that we studied, laparotomy was performed through a lower midline incision and primary repaired with simple closure and apposition of the peritoneum in all operations. There was no doubt that 24 (40.7%) cases of intestinal necrosis or perforation were accompanied by intestinal resection without insertion of the mesh. However, in the other 35 patients, we did not place the mesh, although there was no definite intestinal necrosis or perforation. This is because the intestinal wall edema and inflammation were observed intraoperative due to the long and critical incarcerated intestinal obstruction, and some patients even showed ischemic changes in part of the intestinal wall and had caused secondary peritonitis, which did not rule out delayed intestinal necrosis or intestinal perforation. In addition, it was found in our study that the duration of operation was longer and more blood loss was noted in emergency group, especially in the operation of intestinal resection, and the patients were elderly with critical comorbidities, thus shortening the operation time was also an important factor to be considered. Through short-term follow-up, although 23 patients who accepted emergency surgery were transferred to ICU, and the recurrence rate at 3 years after the operation was 5.1% (3/59 cases) at 3 years after the emergency surgery, most of patients had good therapeutic results and could still enjoy their super-aged lives after surgery. These three patients with recurrence were diagnosed with abdominal CT in our hospital. Finally, they underwent elective surgery with mesh in our hospital and recovered well. It is true that there may be more asymptomatic recurrence that we did not notice and this is one of the limitations of this study. For the patients in the elective surgery group who received primary repaired with mesh, the recurrence rate at 3 years after the operation was 7.1% (1/14 cases), which may be the result of our small sample size (only 14 cases). We followed up this patient with recurrence, who is diagnosed with the treatment of other serious diseases in other hospital, abandoned medical treatment of hernia and died. We did not get accurate information about the cause of recurrence, and we speculated that the diagnosis was unclear or the mesh may have loosened because it was not firmly fixed.

## Conclusion

CT examination plays an important role in improving the diagnostic rate of obturator hernia. In elderly people with comorbidities, timely surgical treatment is the key to improve the efficacy of obturator hernia and prevent the deterioration of the condition. Meanwhile, different surgical methods should be selected according to patients' medical conditions at admission, such as choice of surgical incision and whether to place the mesh or not. Most patients can achieve a good therapeutic effect and enjoy their super-aged lives after primary closure surgery without prosthetic materials, but the recurrence rate may increase. In addition, intestinal resection and postoperative complications may be the important factors leading to postoperative death.

## Data Availability

The datasets used and/or analysed during the current study available from the corresponding author on reasonable request.

## Abbreviations

OH: Obturator hernia
CT: Computed tomography
ICU: Intense care unit
SSI: Surgical site infection
MODS: Multiple organ disfunction syndrome
AECOPD: Acute exacerbation chronic obstructive pulmonary disease
